# Use of Botulinum Toxin for Limb Immobilization for Rehabilitation in Rats with Experimental Stroke

**DOI:** 10.3390/biom13030512

**Published:** 2023-03-10

**Authors:** Hongxia Zhang, Jialing Liu, Deborah Bingham, Adrienne Orr, Masahito Kawabori, Jong Youl Kim, Zhen Zheng, Tina I. Lam, Stephen M. Massa, Raymond A. Swanson, Midori A. Yenari

**Affiliations:** 1 Department of Neurological Surgery, University of California, San Francisco, CA 94143, USA; 2 San Francisco Veterans Affairs Medical Center, San Francisco, CA 94121, USA; 3 Department of Neurology, University of California, San Francisco, CA 94143, USA; 4 Department of Neurosurgery, Hokkaido University, Sapporo 060-0808, Japan; 5 Department of Anatomy, Yonsei University, Seoul 03722, Republic of Korea

**Keywords:** stroke, rehabilitation, rodent, MCAO, constraint induced movement therapy, motor function recovery

## Abstract

Motor rehabilitation strategies after unilateral stroke suggest that the immobilization of the healthy, unimpaired limb can promote the functional recovery of a paretic limb. In rodents, this has been modeled using casts, harnesses, and other means of restricting the use of the non-paretic forelimb in models of experimental stroke. Here, we evaluated an alternative approach, using botulinum toxin injections to limit the function of the non-paretic forelimb. Adult male rats were subjected to permanent ligation of the left distal middle cerebral artery, resulting in right forelimb paresis. The rats were then subjected to: (1) no treatment; (2) botulinum toxin injections 1 day post stroke; or (3) cast placement 5 days post stroke. Casts were removed after 5 weeks, while the botulinum toxin injection effectively immobilized subjects for approximately the same duration. Rats with bilateral forelimb impairment due to the stroke plus casting or botulinum injections were still able to feed and groom normally. Both immobilization groups showed modest recovery following the stroke compared to those that did not receive immobilization, but the casting approach led to unacceptable levels of animal stress. The botulinum toxin approach to limb immobilization had both advantages and disadvantages over traditional physical limb immobilization. The major advantage was that it was far less stress-inducing to the subject animals and appeared to be well tolerated. A disadvantage was that the paresis took roughly 10 weeks to fully resolve, and any degree of residual paresis could confound the interpretation of the behavioral assessments.

## 1. Introduction

Ischemic stroke remains a leading cause of disability in the developed world. Recovering functional mobility following stroke is one of the most important goals for patients in order to continue living an independent life [1]. To date, there are few recognized treatments, although most focus on rehabilitative efforts. One current therapy is known as constraint-induced movement therapy (CIMT), a neurorehabilitative method where the non-paretic limb is immobilized to promote the use of the weakened limb, thus reducing learned non-use. CIMT has been shown to be beneficial in both human and rodent studies [2]. The constraint of upper-extremity function between 3 and 9 months post stroke and traumatic brain injury (TBI) was shown to improve recovery, with CIMT patients showing improved function compared to patients who did not receive CIMT [3,4]. This improved outcome was not restricted to adults and has also been found to benefit brain-injured children, who also saw improved outcomes with CIMT [5,6]. As such, this approach has been modeled in rodent stroke models to show that the immobilization of the unaffected limb has similar salutary properties to that in humans [7].

To date, casting and jacket/cuff approaches are the most commonly used in rodent stroke models. The unimpaired limb is typically cast to the body by wrapping the limb and body in fabric before covering the limb and torso in plaster-of-Paris strips. Another approach is to fit the animal with a sleeveless jacket with a wrist cuff that attaches to the bodice, allowing defined periods of restraint [7]. However, these approaches restrict many normal movements and can impart undue stress to rodents. As an alternative, we compared casting with a new, potentially less stressful approach using botulinum toxin (Botox). Botox A is a paralyzing agent used as a treatment to weaken muscles in conditions such as muscle spasticity following stroke or TBI [8,9,10,11], or facial synkinesis complications in Bell’s palsy [12] or cerebral palsy [13]. Botox has even been used in post-stroke rehabilitation to treat muscle spasticity in combination with CIMT [14].

In this study, we evaluated two different methods of limb constraint in experimental stroke using either physical casting or inducing temporary paralysis by injecting Botox into the unaffected forelimb. We show that the Botox approach is feasible and well tolerated.

## 2. Materials and Methods

### 2.1. Animals, Housing, and General Considerations

All experimental procedures were approved by the San Francisco Veterans Affairs Medical Center Animal Care and Use Committee. Male Sprague–Dawley rats (250–300 g; 8–10 weeks of age from Simonsen Laboratories, Gilroy, CA, USA) were housed, two per cage, on a reverse 12 h dark/light cycle with access to food and water ad libitum. All procedures including surgery, behavioral assessment, and histological quantification were conducted by trainers/examiners blinded to experimental conditions.

### 2.2. Induction of Distal Middle Cerebral Artery Occlusion

Rats were anesthetized using isoflurane with a face mask and maintained with 1.5% isoflurane in 150 mL/min oxygen and 850 mL/min air. A focal cerebral infarct was induced by permanent occlusion of the left middle cerebral artery (MCA) with a temporary occlusion of the bilateral common carotid arteries (CCAs), as described previously [15]. Male SD rats were anesthetized under the above-mentioned conditions. Core temperature was maintained between 36.5 and 37.0 °C throughout the procedures. The bilateral CCAs were exposed through a ventral midline incision of the neck. A vertical skin incision 1.5 cm in length was made between the right eye and ear. The temporal muscle was scraped from the temporal bone, and a 7 × 7 mm temporal craniotomy was performed, using a small dental drill. To prevent CSF leakage, the dura mater was carefully kept intact, and the left MCA was coagulated and cut through the dura mater. Then, the cranial window was closed with the temporal bone flap. The temporal muscle and skin were sutured with 4-0 nylon threads, respectively. Subsequently, the bilateral CCAs were occluded by surgical microclips for 90 min.

### 2.3. Casting Induced Constraint

**Figure 1:**
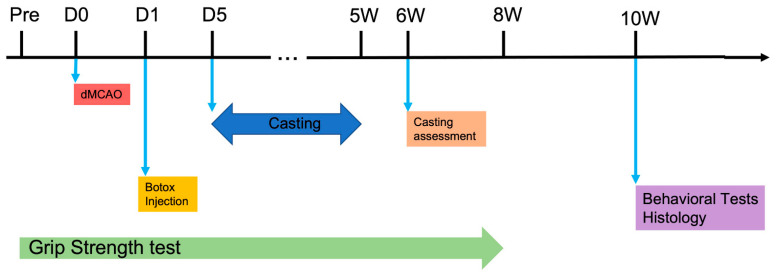
Timeline for experimental design. Botox was administered one day after the induction of stroke via 4 injections, followed by behavioral and histological outcome assessment 10 weeks after stroke. Limbs were restrained via casting from day 5 to 5 weeks after stroke, and behavioral evaluation began at 6 weeks after stroke.

CIMT-treated animals were fitted with an elastic bandaged cast as described [16]. The upper torso was wrapped in soft felt, and the ipsilateral forelimb was wrapped in felt and positioned in a naturally retracted position against the animal’s sternum. Elastic bandage strips were wrapped around the immobilized limb and upper torso for 5 weeks, beginning 5 days after the induction of stroke [16]. CIMT-treated animals were forced to rely on their impaired limbs for movement in standard home cage without daily training. A timeline for treatment and outcome evaluation in relation to the induction of stroke is shown in Figure 1.

### 2.4. Botox-Induced Limb Constraint

To mimic constraint-induced therapy in the rats and enforce the use of the stroke-impaired arm, groups of injured and sham rats received injections of botulinum toxin type A (Botox A) (Allergan, Inc., Irvine, CA, USA), from this point on referred to as Botox. In one group (forearm), animals received injections in the unaffected (left) forelimb one day after the induction of stroke. In this group, a total of 4 muscles (2 in the biceps and 2 in the flexor carpi ulnaris) were each injected with 2 doses in a four-hour interval of 1.25U Botox in 0.05 mL saline. In another group (FLX), animals received similar injections, but only to the left flexor carpi ulnaris.

The effect of Botox or stroke on the muscle strength of the forepaw of the affected or contralateral forepaw was determined via a digital force meter (Chatilon DFE series, MedQuip, Inc., Largo, FL, USA) with a T-bar attachment [17]. Each rat was gently pulled away parallelly from the bar by the tail until it released the bar and the maximum force prior to release of the paw from the bar was recorded. The peak tensile strength (pound per force) from left or right front paw was averaged from 3 consecutive trials prior to and weekly after Botox injection.

### 2.5. Neurobehavioral Assessment

Behavioral outcomes were assessed at different time points due to the longer-lasting effect of the Botox injections. Casting studies were assessed at 6 weeks post stroke, or one week after the casts were removed. The Botox studies were assessed 10 weeks post stroke when the paralyzing effects of the Botox had mostly disappeared. In the Botox groups, grip strength was also assessed weekly for up to 8 weeks. Rats were handled daily for 1 week prior to behavioral testing to acclimate them to test-associated stress (Timeline).

#### 2.5.1. Forelimb Use Asymmetry Test

Animals were placed individually in a Plexiglas cylinder 20 cm in diameter and 30 cm in height and assessed for paw usage during rearing. The number of contacts with the cylinder wall by each paw was determined from 10 min videotaped sessions and limb preference was calculated as a percentage of the independent use of either limb during wall exploration [17,18]. The frequency of contact with both paws was below 3% and thus excluded from the calculation.

#### 2.5.2. Ladder Walk Test

The ladder test is used to assess the forelimb and hindlimb placement from each side along a horizontal ladder that has variable spacing between rungs [17,19]. The rats received 3 trials, which were videotaped for analysis. The number of paw placement faults was recorded for each paw and combined for each side.

#### 2.5.3. Sticky Label Test

Adhesive labels (0.5-inch-diameter round) were placed on the heel of each forepaw. The time to initially sense and then remove the label from each paw was recorded. Five trials were conducted, and the median value was calculated for each rat. Individual trials were timed out at 10 min [17,20].

#### 2.5.4. Vermicelli Handling Test

Rats are known to skillfully manipulate food for eating; thus, the vermicelli handling test is a measure of manual dexterity [21]. Rats were food-restricted for one week to reduce body weight by 10% and were acclimated to eating pasta with small amounts of vermicelli placed in the cages [17,22]. During the test, rats received 5 pieces of dry vermicelli, each 7 cm in length in their home cages, and eating and manipulation of the vermicelli was videotaped for analysis. Normal handling behavior involves holding the vermicelli fragment asymmetrically with 2 paws, one as a guide paw and the other as a grasping paw, with the paws apart until the fragment is approximately 3.5 cm in length. Atypical behaviors in handling the pasta were classified into two categories. Type I atypical behaviors either occurred or did not. These behaviors included: (1) symmetric paw placement when eating the first half of the pasta; (2) hunched body posture while eating; and (3) head is tilted or face is lowered towards the cage floor while eating. Type II atypical behaviors were scored according to the frequency of the occurrence and included: (1) animal uses its mouth to pull the noodle through the forepaws; (2) only one paw is used to manipulate the pasta; (3) the grasp and guide hand switch places; (4) the rat breaks the pasta to small pieces to eat rather than continuously eat the whole piece; and (5) the pasta is dropped or flipped shortly after eating begins.

#### 2.5.5. Computer-Assisted Method for Gait Analysis

Rats were subjected to 3 consecutive runs of gait assessment using the CatWalk automated gait analysis system (Noldus Information Technology, Wageningen, The Netherlands) 10 weeks after stroke or sham surgery. The apparatus consisted of a 1.3 m-long glass plate with dim fluorescent light beamed into the glass from the side. In a darkened environment (below 20 LUX of illumination), the light was reflected downward and the images of the footprints were recorded by the camera under the walkway when the animal’s paws came into contact with the glass surface. The images were processed with a threshold set at 30 arbitrary units from total a. u. of 0 to 225 and analyzed for spatial (print area, print area during maximal contact) and temporal (stride length, swing speed) characteristics of each paw. The print area represents the complete pawprint of all frames that make up a stance, while maximal contact area is the total floor area occupied by the paw during maximal paw contact. Stride length is the distance between two consecutive paw placements of the same paw. Swing speed is the stride length divided by the swing duration when the paw is not in contact with the glass plate [23,24].

### 2.6. Brain Preparation and Histological Analysis

At the end of the observation period, animals were euthanized and perfused, and brains were rapidly removed, frozen, and fixed in 4% PFA. 40 µm-thick brain sections in the coronal plane were prepared and stained with hematoxylin and eosin (H&E). Ischemic lesion sizes were estimated as previously described and expressed as the %ischemic hemisphere [25].

### 2.7. Statistical Analysis and RIGOR

Data were expressed as mean ± SD. Data were analyzed using two-way or multivariate ANOVA with GraphPad v9. Post hoc tests were conducted when appropriate with Bonferroni corrections. Differences between groups were considered significant when *p* < 0.05. Animals were randomized to experimental groups, with randomization being separate for the casting and Botox studies, which were carried out sequentially. Data were further analyzed by investigators not directly involved in the animal experiments.

## 3. Results

### 3.1. Histological Analysis

To verify ischemic infarcts, H&E-stained sections were identified in all cases. Average lesion sizes were similar between all groups (~80 mm^3^), and there were no statistically significant differences between groups. Thus, any difference in neurobehavioral assays cannot be attributed to differences in infarct size.

### 3.2. Behavioral Outcomes

#### 3.2.1. Limb Constraint by Casting Reversed Stroke-Induced Limb Preference

**Figure 2:**
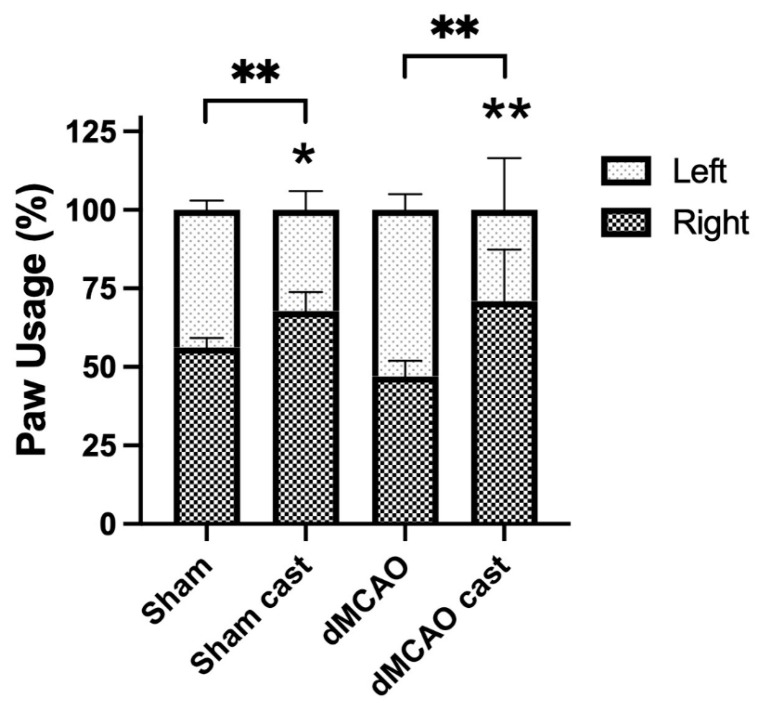
Casting reduced forelimb asymmetry after stroke. Casts were removed 5 weeks post stroke and rats were tested for forelimb asymmetry (cylinder test). Rats receiving sham surgery and casting (Sham cast) and rats receiving stroke and casting (dMCAO cast) showed significant preference for the non-casted (right, impaired) forelimb during wall exploration in the cylinder test, compared to rats that did not receive casting, whether they underwent sham surgery (Sham) or stroke (dMCAO). * *p* < 0.05, ** *p* < 0.01. (*n* = 4/group).

Casting had an overall effect on the forelimb asymmetry (cylinder) test (two-way ANOVA, cast effect: *p* < 0.01). Casted rats without stroke (Sham cast) displayed a significant increase in using the non-casted (right) forelimb compared to sham surgery rats without cast (sham) (*p* < 0.05). Casted rats receiving stroke (dMCAO cast) also preferred using the non-casted, stroke-impaired (right) forelimb compared to MCAO rats without casting (dMCAO). This is in line with prior studies suggesting that casting of the unimpaired limb ultimately improved the use of the impaired limb (Figure 2). Unfortunately, this casting approach to limb immobilization appeared to impart additional stress on the animals, as evidenced by increased porphyrin secretion from the eyes. Thus, an alternative method of limb mobilization was considered.

#### 3.2.2. Botox Had a Sustained Effect on Weakening the Unaffected Forelimb

In light of the debilitating effect of casting, we explored an alternative limb-constraint method using Botox injection. Unlike our experience with casting, no animal in these studies demonstrated stress or increased porphyrin secretion. The Botox appeared to be well tolerated in all cases.

**Figure 3:**
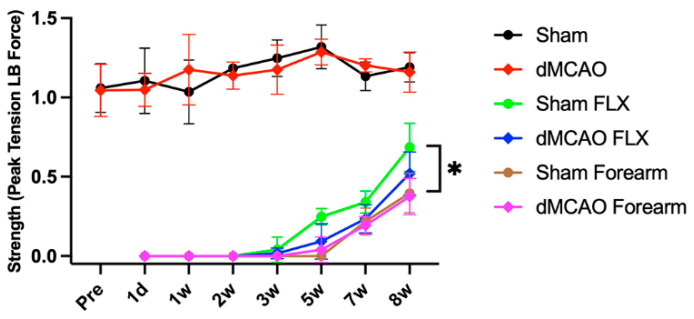
The effect of Botox on left forepaw grip strength. Botox immediately and completely obliterated grip strength of the injected left forepaw. Three weeks after injection, the groups receiving Botox in the flexor (Sham FLX and dMCAO FLX) began to recover, reaching 40–50% of pre-injection strength at 8 weeks. Rats with Botox injected into the forearm (Sham Forearm) displayed a delayed and lesser recovery of grip strength compared to those injected into the flexor (Sham FLX). There was no difference in grip strength in the right forelimb between vehicle-treated sham (Sham) and vehicle-treated stroke (dMCAO) rats. * *p* < 0.05. (*n* = 4–12/group).

Grip strength was determined in the left and right forelimbs to monitor the effect of Botox and stroke in each forelimb prior to and following vehicle or Botox injections in the stroke or sham rats. There was no significant difference in grip strength in the left (unaffected) forelimbs of rats receiving saline injection between stroke and sham groups, indicating that the stroke itself did not reduce the strength of the left (contralesional) forelimb (Figure 3). All rats receiving Botox injection immediately lost grip strength in the left (unimpaired) forelimb compared to vehicle-treated rats (two-way ANOVA, Botox effect: *p* < 0.001; time effect: *p* < 0.001). eight weeks after Botox injection, there was a 40–50% recovery of the grip strength. Rats with Botox injected into only the flexor carpi ulnaris muscles (FLX) displayed an earlier and increased recovery of grip strength compared to those injected in the forearm (two-way ANOVA: effect of Botox location: *p* < 0.001; time effect: *p* < 0.001) (Figure 3). The extent of recovery was significantly improved in the FLX group compared to the forearm-injected sham group (no stroke) rats (*p* < 0.05). To avoid the stress related to prolonged weakening of the contralesional limb, the flexor-injection paradigm was used for the remainder of the study.

#### 3.2.3. Effect of Limb Immobilization by Botox in Experimental Stroke

**Figure 4:**
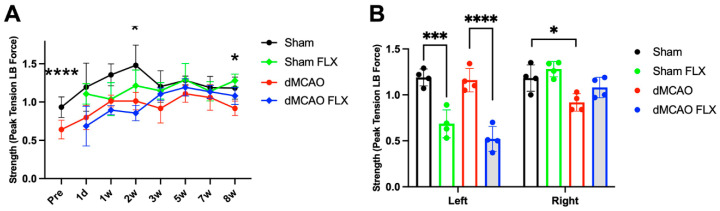
Botox reduced grip strength of the injected left forelimb but improved grip strength of the right forelimb in rats 8 weeks after treatment. (**A**) Grip strength of the right limb over 8 weeks. Stroke rats had significantly reduced grip strength in affected limb prior to injection of Botox. In the vehicle-treated groups, significant difference in grip was detected between stroke and sham rats at 2 wks and 8 wks following treatment. Three-way ANOVA revealed a main effect of stroke or Botox on the recovery of grip strength over 8 wks of time. (**B**) At 8 wks after injection, Botox reduced grip strength in the left forelimb, while stroke reduced strength in the right forelimb. Although there was an overall effect of Botox in improving the grip strength in the right forelimb, it did not reach significant difference between the two stroke groups (dMCAO vs. dMCAO FLX). * *p* < 0.05, *** *p* < 0.005, **** *p* < 0.001. (*n* = 4/group).

Limb immobilization by Botox improved the grip strength of the contralateral limb following experimental stroke and amongst sham controls. Prior to Botox injection, the right (impaired) paw of stroke mice had decreased grip strength compared to the sham uninjured group (*p* < 0.0001, Figure 4A). Botox immobilization also led to improved recovery of grip strength over the 8-week course of treatment for both the sham and stroke groups (three-way ANOVA, stroke × time effect: *p* < 0.05; Botox × time effect: *p* < 0.01). By 8 weeks, the right, impaired forelimb of the stroke group not receiving Botox still had reduced strength compared to the sham counterpart (Figure 4B), but there was no difference in grip strength between the Botox-treated stroke group and the uninjured shams. While there was no difference between the two stroke groups (Botox vs. no Botox), stroke animals receiving Botox had grip strength closer to that of the shams, and might suggest that Botox immobilization of the uninjured forelimb may lead to improved strength of the impaired limb. There was also an overall effect of Botox in reducing grip strength of the left forelimb, which would be expected considering the paralyzing effects of the drug (two-way ANOVA, Botox effect: *p* < 0.0001). Interestingly, Botox slightly improved the strength of the right forelimb in both sham and stroke rats (two-way ANOVA, Botox effect: *p* < 0.05) (Figure 4B).

#### 3.2.4. Botox Reduced Stroke-Induced Forelimb Asymmetry

**Figure 5:**
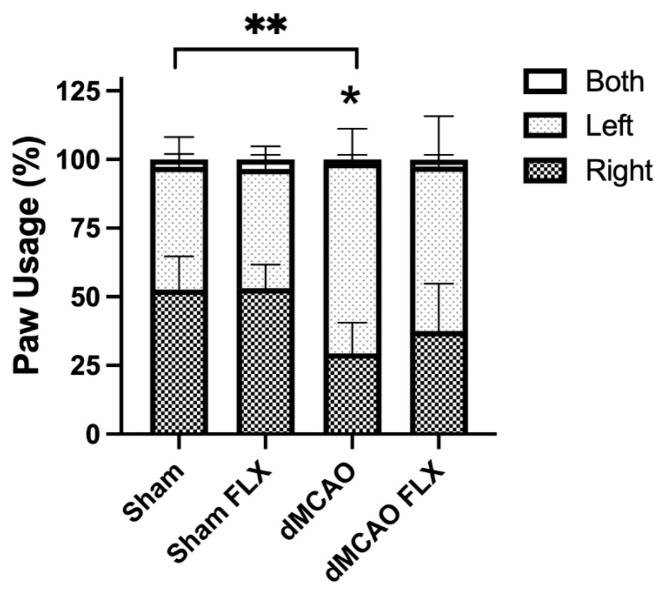
Botox led to reduced forelimb asymmetry after stroke. Vehicle-treated (Sham) or Botox (Sham FLX)-treated rats exposed to sham surgery showed no preference for either forelimb during wall exploration on the cylinder test, while vehicle-treated rats subjected to stroke (dMCAO) displayed a significant preference for the unimpaired forelimb. Unlike the vehicle-treated stroke rats, Botox-treated stroke rats (dMCAO FLX) no longer exhibited preference of paw usage. * *p* < 0.05, ** *p* < 0.01. (*n* = 11/group). The left-sided graph shows only single-paw contacts to the cylinder, while the right-sided graph includes instances where both paws contacted the cylinder.

There was a significant effect of stroke, but not Botox, in the forelimb asymmetry (cylinder) test (two-way ANOVA, stroke effect: *p* < 0.0005; Botox effect: *p* = 0.259). In the sham groups, rats subjected to stroke (dMCAO) displayed significant forelimb asymmetry compared to sham rats (*p* < 0.001). Among groups receiving Botox, rats exposed to stroke that received Botox (dMCAO FLX) did not show significant preference in forelimb usage compared to those that received vehicle (dMCAO) (Figure 5), suggesting that Botox improved the recovery of the impaired forelimb. The total number of wall explorations did not differ significantly between groups, suggesting that neither stroke nor Botox affected exploratory activity.

#### 3.2.5. No Effect of Limb Immobilization by Botox and Sensorimotor Function

**Figure 6:**
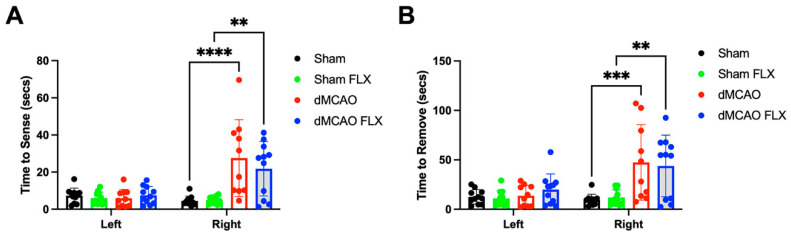
Botox did not significantly reduce the sensorimotor dysfunction in sticky-label removal after stroke. Stroke rats treated with vehicle (dMCAO) or Botox (dMCAO FLX) took more time to sense (**A**) and remove (**B**) sticky tape in the right paw, compared to their counterparts subjected to sham surgery (Sham, Sham FLX). ** *p* < 0.01, *** *p* < 0.005, **** *p* < 0.0001. (*n* = 10–11/group).

The adhesive tape removal test assesses both sensory and motor functions, which can be quantified by the time it takes to initially contact or sense the adhesive on the paw (sensory) and the time that it takes for the rodent to remove the adhesive (motor). Animals receiving stroke required more time to sense the adhesive, but Botox treatment had no effect on this. This is not surprising since limb immobilization would not be expected to improve sensation. However, time to adhesive removal was not improved among animals that were treated with Botox (two-way ANOVA, Time to sense: stroke effect: *p* < 0.0001, Botox effect: *p* = 0.4976; Time to remove: stroke effect: *p* < 0.0001; Botox effect: *p* = 0.9387). Thus, Botox did not significantly improve stroke-induced sensorimotor function. Interestingly, Botox injected into the left forelimb did not affect the detection or removal of sticky tape on the left paw (Figure 6). Not surprisingly, there was no significant effect of stroke on the ability to sense and remove adhesives on the non-impaired left paw.

#### 3.2.6. Botox Did Not Improve Post-Stroke Motor Impairment in Ladder Walking

**Figure 7:**
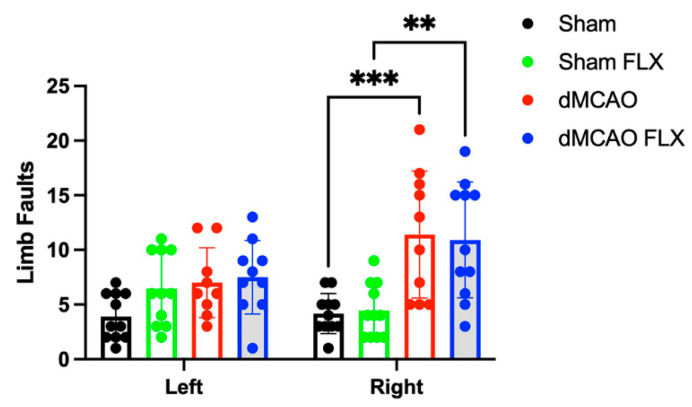
Botox did not significantly reduce the frequency of limb faults on the affected side in the horizontal ladder after stroke compared to vehicle treatment. Vehicle-treated stroke (dMCAO) rats displayed significantly more limb faults on the affected side compared to vehicle-treated rats that underwent sham surgery (Sham). In the Botox treatment groups, stroke rats (dMCAO FLX) showed significantly increased limb faults in affected limbs compared to those that underwent sham surgery and also received Botox (Sham FLX). However, the number of limb faults did not differ between Botox-treated stroke rats (dMCAO FLX) and vehicle-treated stroke rats (dMCAO). ** *p* < 0.01, *** *p* < 0.005. (*n* = 10–11/group).

There was a significant effect of stroke, but not Botox, on the number of limb faults when traversing a horizontal ladder (two-way ANOVA, stroke effect: *p* < 0.0001; Botox effect: N.S.) (Figure 7). Vehicle-treated stroke (dMCAO) rats displayed significantly more foot faults on the affected side than vehicle-treated rats subjected to sham surgery (Sham) (*p* < 0.005). In the Botox treatment groups, stroke rats (dMCAO FLX) showed significantly increased foot faults in the affected limb than rats with sham surgery (Sham FLX) (*p* < 0.01), but they did not perform significantly better compared to vehicle-treated stroke rats (dMCAO) (N.S.), suggesting that Botox did not improve motor deficits as assessed by the horizontal ladder test. There was no significant effect of stroke or Botox in the number of limb faults of the unaffected side during the ladder test (N.S.). This is not particularly surprising, since faults from both fore and hindlimbs were counted. Since forelimb immobilization is not expected to improve hindlimb function, improvements from Botox treatment in this measure may have been diluted by including hindlimb faults.

#### 3.2.7. Limb Immobilization by Botox and Fine Motor Skills

**Figure 8:**
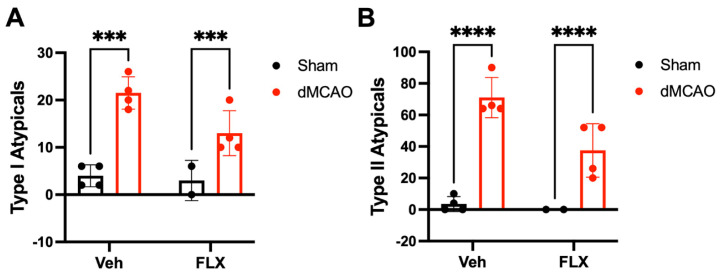
Botox did not significantly reduce atypical behaviors in the vermicelli handing test among stroke rats. Stroke rats treated with vehicle (dMCAO Veh) or Botox (dMCAO FLX) showed significantly increased type I (**A**) and type II (**B**) atypical behaviors, compared to those that underwent sham surgery regardless of treatment. Despite an overall effect of Botox in reducing type I and type II atypical behaviors, between-group comparisons did not show statistical significance between the stroke groups. *** *p* < 0.005, **** *p* < 0.001. (*n* = 4/group).

Stroke increased the atypical behaviors during vermicelli consumption, while Botox reduced them (two-way ANOVA, stroke effect: *p* < 0.0001; Botox effect: type I typical: *p* < 0.05; type II atypical: *p* < 0.05). Both vehicle (dMCAO)- and Botox-treated stroke rats (dMCAO FLX) displayed significantly greater numbers of type I (Figure 8A) and type II (Figure 8B) atypical behaviors over five trials of pasta tests compared to their sham counterparts (Sham and Sham FLX). While there was a trend towards reduction in both type I and type II atypical behaviors in Botox-treated stroke rats compared to vehicle-treated stroke rats, inter-group comparisons did not reach statistical significance (Atypical I, *p* = 0.1872; Atypical II, *p* = 0.0998).

#### 3.2.8. Stroke and Botox Both Affected the Spatial and Temporal Parameters of Paw Placement during Catwalk Test

**Figure 9:**
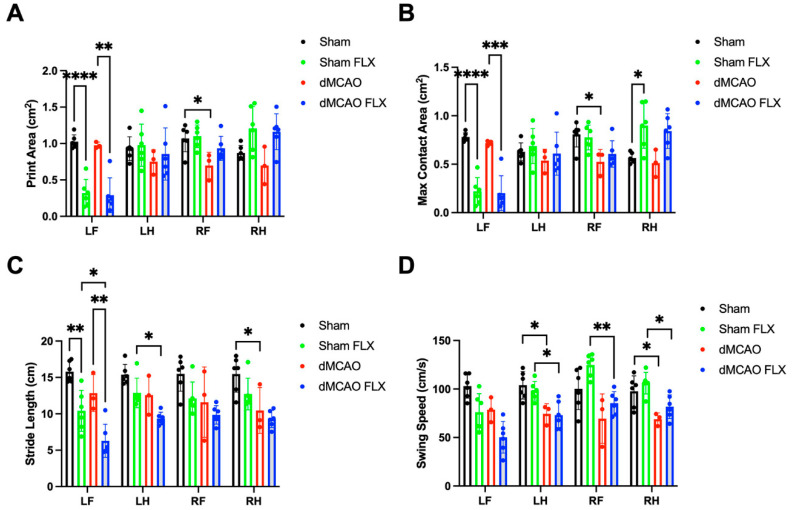
Botox did not significantly improve spatial or temporal characteristics of paw placement during catwalk test in stroke rats. Stroke rats treated with vehicle (dMCAO) had a significantly reduced print area (**A**), maximal contact area (**B**), stride length (**C**), and swing speed (**D**) in the affected limbs during catwalk test compared to vehicle-treated sham rats. Likewise, Botox reduced print area (**A**), maximal contact area (**B**), and stride length (**C**) in left forelimb (LF) in rats with sham surgery (Sham FLX) or stroke (dMCAO FLX) compared to their vehicle-treated counterparts. However, the spatial and temporal parameters of paw placement did not differ between Botox-treated stroke rats (dMCAO FLX) and vehicle-treated stroke rats (dMCAO). * *p* < 0.05, ** *p* < 0.01, *** *p* < 0.005, **** *p* < 0.001. (*n* = 3/dMCAO group and *n* = 6/other group).

Two-way ANOVA revealed a significant effect of stroke in reducing the pawprint area during stance as well as print area at maximal contact in the right forepaw (RF) of rats with stroke (*p* < 0.005), and a significant effect of Botox in decreasing the size of the left forepaw (LF, *p* < 0.0001) and right hindlimb (RH, *p* < 0.001). Botox did not significantly increase print area or maximum contact area in rats with stroke (Figure 9A,B).

There was a significant reduction in the distance between successive placements of each paw during maximal contact, known as stride length (two-way ANOVA, stroke effect: LF:*p* < 0.005; LH: *p* < 0.0005; RF: *p* < 0.05; RH: *p* < 0.001). A significant Botox effect was seen in LF (*p* < 0.0001), LH (*p* < 0.001), and RF (*p* < 0.05). However, Botox did not significantly improve stride length in rats with stroke (Figure 9C).

Stroke reduced the swing speed of all four paws during walking due to reduced stride length and prolonged swing duration (two-way ANOVA, stroke effect: *p* < 0.005/LF; *p* < 0.0005/LH; *p* < 0.005/RF; *p* < 0.0005/LF), while Botox reduced the swing speed of the front paws (Botox effect: *p* < 0.005/LF; *p* < 0.05/RF). Botox did not significantly enhance stroke-impaired swing speed (Figure 9D).

## 4. Discussion

We show a new approach to limb immobilization in our rodent stroke model using Botox that can be readily applied to approximate limb immobilization strategies in stroke patients. Prior studies have used a casting approach which, in our hands, led to unacceptable levels of stress. In contrast, limb immobilization by Botox seemed well tolerated and immobilization through injection of only the forelimb flexor muscles led to transient paralysis that was sustained for approximately 8 weeks. This could be an effective method to model this rehabilitation strategy in experimental models.

The purpose of this study was largely explorational, to determine whether this approach was feasible in this rodent stroke model. As such, experimental numbers were low for some assessments, and only trends could be observed. This was the case for the casting studies, where it was no longer possible to continue with this approach, and further studies needed to be abandoned. The studies with Botox treatment first established the optimal dose and location of the injections. The group that received four injections into the forearm led to too much paralysis that required more than 8 weeks to recover. While this paralysis seemed well tolerated, the duration and severity of the paralysis made it difficult to carry out many of the neurobehavioral studies, particularly during timepoints typically prespecified in rodent stroke studies. The group that received injections to only the flexor carpi ulnaris experienced moderate weakness that recovered well enough that neurobehavioral studies could be performed. While there was still the potential that the residual weakness from the Botox could have confounded our motor assessments, we were still able to detect a suggestion of benefit from Botox treatment.

Most of our positive results could be seen in studies that assessed forelimb motor function such as the grip strength, cylinder, and vermicelli tests. The lack of differences, particularly in the adhesive removal, ladder, and catwalk tests, may have more to do with these being assays that also measure sensory and hindlimb function, neither of which forelimb immobilization would be expected to improve. It is also possible that some of the trends observed reflect a type II statistical error due to the small number of experiments for some of the assays. While our study only focused on forelimb immobilization, lower-limb immobilization has also been studied at the clinical level in stroke and related conditions [26]. Clinical studies of lower-limb immobilization have claimed to improve leg function, although whether this necessarily improves balance and gait speed has been debated. Regardless, this approach could also be applied in animal models where hind-limb muscles could be temporarily immobilized with Botox. Future studies might focus on lower-limb constraint therapy in animal models.

It also bears mentioning that limb immobilization alone is an incomplete therapy. At the clinical level, CIMT is used only during periods of rehabilitation and includes physical therapy. In this exploratory feasibility study, we did not add physical therapy, which would normally be needed in order to optimize the benefit of constraint therapy. Instead, rodents were allowed to move freely in their home cages, but were not provided additional stimuli to encourage rehabilitation. In a prior study from our group, where animals subjected to experimental TBI and given both Botox forelimb immobilization and physical therapy consisting of environmental enrichment and coaching by investigators showed improvement compared to animals that that received neither [17]. Thus, we are optimistic that had we included physical therapy in the current study, we should have been able to show a benefit of this approach. Clearly, future studies should include physical therapy in order to show optimal benefit. However, we were still able to show some trends favoring constraint therapy, even without physical therapy. This approach could also be used to test combination restraint therapy plus other pharmacological approaches. Since rehabilitation remains a key approach to neurological recovery following ischemic stroke, these lines of investigations should be encouraged.

We would also point out that we do not suggest that this approach be used in humans. There are already effective methods of restraining the functional limb for defined periods during supervised physical therapy without having to paralyze the good limb continuously with Botox. To attempt this in humans could lead to additional disability and harm outside of supervised therapy. Thus, this model should be seen as a correlation to the already established clinical settings where physical restraint of the unaffected limb enhances the recovery of the paretic limb. Our own experience shown here in rodents indicates that pharmacological restraint is less stressful to rodents. We suggest that when modeling constraint therapy in experimental stroke, particularly in models using quadrupeds such as rodents, limb immobilization using Botox or a similar paralyzing agent may be preferable to physical constraint.

## Data Availability

Not applicable.
